# Metabolic profiling and gene expression analysis reveal the quality deterioration of postharvest toon buds between two different storage temperatures

**DOI:** 10.3389/fpls.2023.1142840

**Published:** 2023-03-20

**Authors:** Hu Zhao, Cheng Shen, Qingping Hao, Mingqin Fan, Xiaoli Liu, Juan Wang

**Affiliations:** Biology and Food Engineering College, Fuyang Normal University, Engineering Technology Research Center of Anti-Aging Chinese Herb, Fuyang, Anhui, China

**Keywords:** metabolomics, gene expression, quality deterioration, storage temperature, *toona sinensis*

## Abstract

Toon buds, a popular woody vegetable, contain large amounts of nutrients. However, toon buds have strong respiratory metabolism after harvest and are highly prone to decay, resulting in quality deterioration. Low temperature can effectively inhibit postharvest senescence of toon buds. GC-TOF-MS combined with quantitative real-time PCR was used to elucidate the toon bud deterioration mechanism after harvest by analyzing the difference in the relative contents of primary metabolites and their derivatives, and the expression of key genes associated with metabolic pathways in toon buds between low temperature and room temperature storages for 72 h. Results showed that the ethylene synthesis in toon buds accelerated under room temperature storage, along with significant changes in the primary metabolic pathway. The catabolism of amino acids, fatty acids, and cell membrane phospholipids was accelerated, and the gluconeogenesis synthesis was strengthened. Moreover, the sucrose synthesis was increased, the glycolysis and TCA cycle were broken down, and the pentose phosphate pathway was vigorous. As metabolic intermediates, organic acids were considerably accumulated. Moreover, varieties of toxic compounds were produced in parallel with the activation of aromatic compounds. This work provided a comprehensive understanding of the metabolic regulation, thereby revealing how low and room temperatures differentially influenced the quality deterioration of postharvest toon buds.

## Highlights

- Ethylene production and respiration rate in postharvest toon buds were accelerated.
- A total of 305 metabolites were detected in the metabolic data.
- Sucrose and organic acids were accumulated in postharvest toon buds.
- Catabolism of amino acids was strengthened in postharvest toon buds.
- Some key genes were differentially expressed between two storage temperatures.

## 1 Introduction

*Toona sinensis*, also called Xiangchun in Chinese, is an important woody vegetable widely planted in Asian countries. In food production, toon buds are used as raw materials for sauce and functional food by consumers because of their bright color, distinctive flavor, and multiple nutrients (Jiang et al., 2019; Su et al., 2020).

As a woody vegetable, toon buds are susceptible to decay and quality deterioration during postharvest storage because of their high respiration rate and water content (Zhao et al., 2018; Lin S. H. et al., 2019). Many preservative methods, such as modified atmosphere, film coating, and various chemical reagent treatments, have been employed to prolong the shelf life of toon buds. However, cold storage has been considered as an effective and economical method (Zhang et al., 2009; Yang et al., 2011; Zhu and Gao, 2017). LT contributes to decreasing respiration, slowing metabolism and maintaining the quality of toon buds (Wang et al., 2019). However, the quality of toon buds inevitably deteriorates with the increasing storage time (Hu et al., 2019). Nevertheless, two storage temperature models, i.e., room temperature (RT) and LT, provide an ideal model for deeply analyzing the molecular mechanism of quality deterioration of toon buds. Previous studies have mainly investigated the secondary metabolites in postharvest toon buds. These metabolites determine the color and flavor quality of toon buds (Yang et al., 2011; Yang et al., 2019). After cold storage, the total content of flavonoids and volatile terpenoids and their oxidates increase significantly with prolonged storage time (Zhao et al., 2019; Zhao et al., 2021). However, how the main carbohydrates, organic acids, amino acids, fatty acids, and their metabolic mechanism change in postharvest toon buds during storage remains largely unknown.

Recently, a large number of literatures have reported the physiological, biochemical, and molecular regulatory mechanisms of quality deterioration and postharvest senescence of fruits and vegetables (Tang et al., 2016; Guo et al., 2019; Pott et al., 2020). Tang et al. (2016) revealed that RT storage enhanced ABA and ethylene signaling pathways *via* the upregulation of *PYLs*, *ABI5*, and *ERFs* on Powell orange pulp senescence. The RT-stored upregulated genes were involved in primary metabolism including sucrose metabolism, glycolysis, gluconeogenesis, and fermentation pathways, resulting in declining levels of sucrose and organic acids, such as malate, citrate, and α-ketoglutaric acid, and accumulated hexoses on Powell orange pulp. Postharvest fruit respiration directly affects primary metabolic pathways, including glycolysis and the tricarboxylic acid cycle (TCA), which account for changes in sugar, amino acid, and organic acid levels (Zhang et al., 2011; Goulas et al., 2015). Guo et al. (2019) reported that the metabolic pathways related to carbohydrates, organic acids, and amino acids might be highly active in RT-stored litchi pulps, whereas the metabolic pathways related to aliphatic metabolites and nucleotides might be highly active in LT-stored litchi pulps. The genotype tomato with malate dehydrogenase (MDH) deficiency showed higher malate content and poorer postharvest behavior than non-transformed fruits (Osorio et al., 2019). This finding indicates malate’s role in postharvest responses to RT storage. In the present study, the metabolites in the toon buds at harvest (0 h) were determined through gas chromatograph coupled with a time-of-flight mass spectrometer (GC-TOF-MS) after storage at RT and LT for 12, 24, 48, and 72 h. The DEMs of toon buds after 12, 24, 48, and 72 h of storage at RT and LT were identified compared with those at harvest 0 h (control). Furthermore, the differential expression of the critical genes involved in the primary metabolism and ethylene signaling pathway was revealed between two storage temperature models. In summary, our study shed light on the differential metabolites and candidate genes associated with the quality deterioration of toon buds in both storage models.

## 2 Materials and methods

### 2.1 Plant materials

Freshly harvested toon buds of the ‘Heiyouchun’ cultivar were obtained from the *T*. *sinensis* nursery base in Xin Town of Taihe County in Anhui Province, in April 2021. More than 300 toon buds with uniform color and size but without visual blemishes and mechanical damage were selected. The harvested toon buds collected in ice boxes were immediately transported to the laboratory (control). Then, they were randomly divided into two groups. One group was placed in a hermetic plastic container at RT (20°C ± 0.5°C) with a relative humidity 80%–90%. In comparison, the other group was stored in a refrigerator (4°C ± 0.5°C) for the LT storage with the same humidity. The sampling time points at both treatments were set at 12, 24, 48, and 72 h after storage. Six biological replicates were designed with five toon buds each. The samples were collected at each time point for respiration rate and ethylene content measurements. The quick-freeze samples treated with liquid nitrogen were stored at −80°C and employed for subsequent metabolites and RNA extraction.

### 2.2 Toon bud appearance evaluation

Toon bud appearance was evaluated as previously described by Zhao et al. (2018). The decay symptoms after storage were visually determined as injury ranks using a five-grade marking system based on the percentage of brown leaves or shoots: 0 = intact buds without brown or rotten tissue; 1 = 1% to 25% of the bud-damaged tissue; 2 = 26% to 50% of the bud-damaged tissue; 3 = 51% to 75% the bud-damaged tissue; 4 ≥ 76% of the bud-damaged tissue. The decay index (DI) was calculated following the formula described by Zhao et al. (2018).

### 2.3 Determining respiration rate and ethylene production

Respiration rates were measured according to the modified small-skep-method (MSSM) (Li et al., 2015). Three toon buds with uniform size from each sample were kept in a 0.5-l airtight wide-mouth bottle. The released CO_2_ from postharvest toon buds was absorbed using Ba(OH)_2_ solution, and the residual Ba(OH)_2_ was titrated with oxalic acid solution. The amount of CO_2_ could be calculated from the difference between the oxalic acid solution consumed by the blank and the samples. The result was expressed as mg kg^−1^ h^−1^ of CO_2_. Ethylene production was measured *via* gas chromatography (GC). The samples were placed in 0.5-l airtight plastic bottles (each containing three toon buds) for five time points at both storage temperatures. Then, 0.1 ml of the headspace gas was injected into an Agilent 7890A GC (Agilent, Santa Clara, CA, USA) equipped with a HP-5MS packed column and a flame ionization detector. The amount of ethylene production was calculated from a calibration curve of standard ethylene gas and expressed as ng kg^−1^ s^−1^ of fresh weight.

### 2.4 Non-target GC–TOF-MS analysis

#### 2.4.1 Sample preparation and extraction

Hydrophilic metabolites were extracted from 50 mg of the powered toon buds by adding 500 μl of 3:1 (v/v) methanol: ddH_2_O as a precold extraction mixture. Then, 10 µl ribitol (0.5 g l^−1^ stock solution) was added to the extraction mixture as the internal standard (IS). The extraction was mixed using a thermomixer compact (Eppendorf AG, Germany) for 30 s. A steel ball was added to the extraction to extract the metabolites fully. Moreover, the sample was treated with a 45-Hz grinding instrument for 4 min and ultrasonicated with an ice water bath for 5 min (repeated three times). After centrifugation at 4°C for 15 min at 12,000 rpm, 300 μl supernatant was transferred to a fresh tube. Then, 100 μl of each sample was collected, pooled, and evaporated in a vacuum concentrator to prepare the quality control (QC) sample. The dried samples were redissolved in 80 μl of 20 g l^−1^ methoxyamine hydrochloride in pyridine and incubated at 80°C for 30 min while shaking. Then, derivatization of the mixture was achieved through incubation with 100 μl of BSTFA regent (1% TMCS, v/v) at 70°C for 1.5 h. After the samples were gradually cooled to RT, 5 μl of FAMEs (in chloroform) was added to the QC sample. Then, all samples were subjected to Agilent 7890 GC coupled with a time-of-flight mass spectrometer (GC-TOF-MS) for analysis.

#### 2.4.2 Injection parameters on GC-TOF-MS

A volume of 1 μl aliquot of derivatized sample was injected in splitless mode and separated on a 30 m × 0.25 mm DB-5MS capillary column coated with a 0.25-µm CP-Sil 8 CB low bleed (Varian Inc., Palo Alto, CA, USA). Helium was used as the carrier gas. The front inlet purge flow was 3 ml min^−1^. The gas flow rate through the column was 1 ml min^−1^. The initial temperature was kept at 50°C for 1 min. Then, it was raised to 310°C at a rate of 10°C min^−1^ and kept for 8 min at 310°C. The injection, transfer line, and ion source temperatures were 280°C, 280°C, and 250°C, respectively. The energy was −70 eV in the electron impact mode. The mass spectrometry data were acquired in the full-scan mode with the 50–500-m/z range at 12.5 spectra per second after a solvent delay of 6.27 min.

#### 2.4.3 Metabolite analysis by GC-TOF-MS

Raw data analysis, including peak extraction, baseline adjustment, deconvolution, alignment, and integration, was completed with ChromaTOF (V 4.3x, LECO) software. The LECO-Fiehn Rtx5 database was used for metabolite identification by matching the mass spectrum and retention index (Kind et al., 2009). Finally, the peaks detected in less than half of the QC samples or relative standard deviation (RSD) in>30% of the QC samples was removed (Dunn et al., 2011).

### 2.5 RNA isolation and quantitative real-time PCR analysis

The relative expression levels of the genes selected were analyzed following the method by Zhao et al. (2017) with slight modifications. The total RNA was isolated from 100 mg of toon bud samples with RNAprep Pure Plant Plus Kit (polysaccharide- and polyphenolics-rich) (Tiangen, Beijing). Then, it was reversely transcribed into single cDNA at 42°C for 15 min by using FastKing gDNA Dispelling RT SuperMix (Tiangen, Beijing) according to the manufacturer’s protocols. Then, the cDNA was diluted 10-fold as templates for the quantitative real-time PCR (qRT-PCR) analysis *via* SuperReal PreMix Plus (SYBR Green) (Tiangen, Beijing) performed by the Bio-Rad, CFX96 Connect Optics Module Real-Time System (Thermo, CA). The *actin* gene of *T. sinensis* was used as the internal control for evaluating the relative expression of the specific genes. The gene-specific primers used are listed in **Supplementary Table S1** (Zhao et al., 2017). The expression levels of critical genes involved in the primary metabolism and ethylene signaling were analyzed by qRT-PCR. The 2^–△△^Ct method was applied to calculate the relative expression levels of these genes (Zhao et al., 2017). Each sample also contained three biological replicates and three technological replicates. The relative expression levels were represented by mean ± standard error of the mean (SEM) values (Zhao et al., 2017).

### 2.6 Statistical analysis

The data of metabolites presented in this study were the mean values of six biological replicates. The GC-TOF-MS data were processed with SIMCA software (Version 15.0, Umetrics, Umea, Sweden) to obtain the result of multivariate statistical analysis. Principal component analysis (PCA) was performed to compare the DEM level between both samples. Then, DEMs were identified and confirmed between the two samples using the orthogonal partial least square discriminant analysis (OPLS–DA) with the threshold value of variable importance in projection (VIP) >1 and *P* < 0.05. Subsequently, the expression patterns of DEMs were demonstrated by hierarchical clustering analysis (HCA) and heat maps. The physiological data and qRT-PCR analysis were the mean values of three replicates. A two-way ANOVA was performed. The Fisher test (*P* < 0.05) was used for mean comparisons between the two samples using SPSS 22.0. The different letters between means were indicative of significant difference at a significant level of P < 0.05.

## 3 Results

### 3.1 Alleviation of postharvest senescence and rotting of toon buds by LT

**Figure 1:**
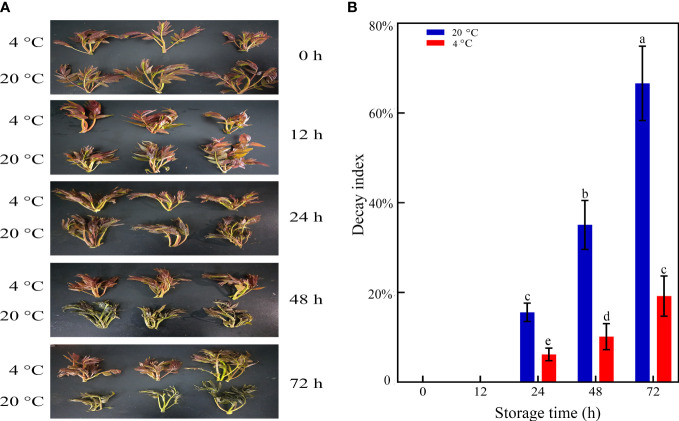
Appearance **(A)** and DI **(B)** of toon buds at 4°C and 20°C storage after 0, 12, 24, 48, and 72 h. The values are presented as the mean ± SEM. The bars with different lowercase letters are significantly different at *P* < 0.05.

Toon bud browning is a common problem affecting quality and consumer preference. The visual effects of LT storage on delaying toon bud senescence, browning, and rotting are shown in **Figure 1A**. The toon buds under LT storage still retained a green appearance after 72 h. In comparison, the toon buds stored in RT developed severe browning and rotting. As illustrated in **Figure 1B**, the DI of the LT-stored toon buds was 17.8% in 72 h, significantly lower than that of RT-stored toon buds (68.7%).

### 3.2 Inhibition of ethylene production and respiration rate in postharvest toon buds by LT

**Figure 2:**
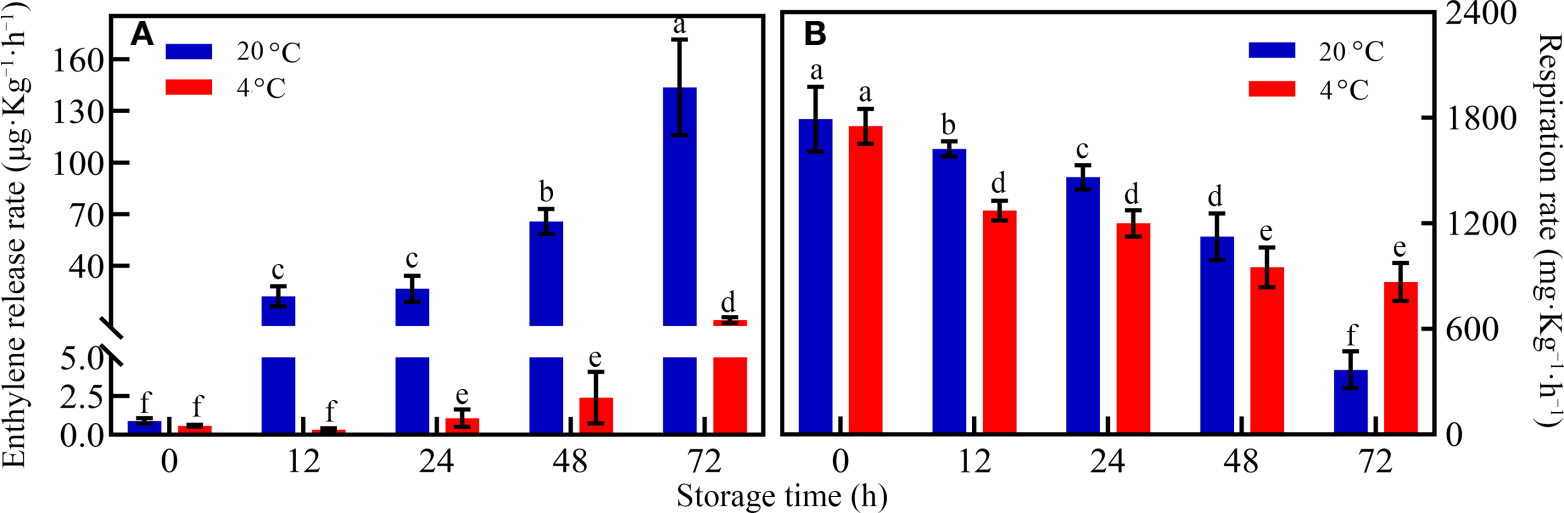
Change in the **(A)** ethylene release rate and respiration rate **(B)** of toon buds at 4°C and 20°C storage after 0, 12, 24, 48, and 72 h. The values are presented as the mean ± SEM. The bars with different lowercase letters are significantly different at *P* < 0.05.

When the storage temperature regime was not considered during the whole storage period, the ethylene concentrations increased with prolonged storage time (**Figure 2A**). However, the ethylene release rate significantly differed between RT and LT storages. When toon buds were under LT conditions, the ethylene release rate was very low during the whole storage period. The ethylene release rate was only 2.34 ng kg^-1^ s^-1^ after 72 h of storage. However, the ethylene release rate increased exponentially after a relatively stable period of 24–48 h under RT storage. The ethylene release rate reached 39.90 ng kg ^−1^ s ^−1^ at the end of the storage period (**Figure 2A**).

Unlike the ethylene release rate, the respiration rate demonstrated various downward trends in both groups (**Figure 2B**). During 0–48 h of RT storage, the respiration rate of the LT storage was lower than that of the RT storage. The finding indicated that the respiratory consumption of toon buds was inhibited under LT conditions. After 72 h, the respiration rate in RT was lower than that in LT.

### 3.3 Identification of the metabolites in postharvest toon buds

A total of 305 metabolites were detected in the raw data, of which 150 matched the known metabolites in the database, including carbohydrates, organic acids, amines, aldehydes and alcohols, amino acids, fatty acids, alkaloids, pyridines and purines, and their derivatives. The remaining 155 metabolites were named as “unknown” or “analyte.” Their structural features needed to be further identified (**Supplementary Table S2**). The correlation coefficients (six biological duplicate samples) within a group exceeded a threshold value (0.7) within the range of 0.77−0.98. This finding suggested that the data within the group showed good repeatability and could be used for subsequent differential metabolite screening (**Supplementary Table S3**).

### 3.4 Multivariate analysis of metabolomic data in postharvest toon buds

**Figure 3:**
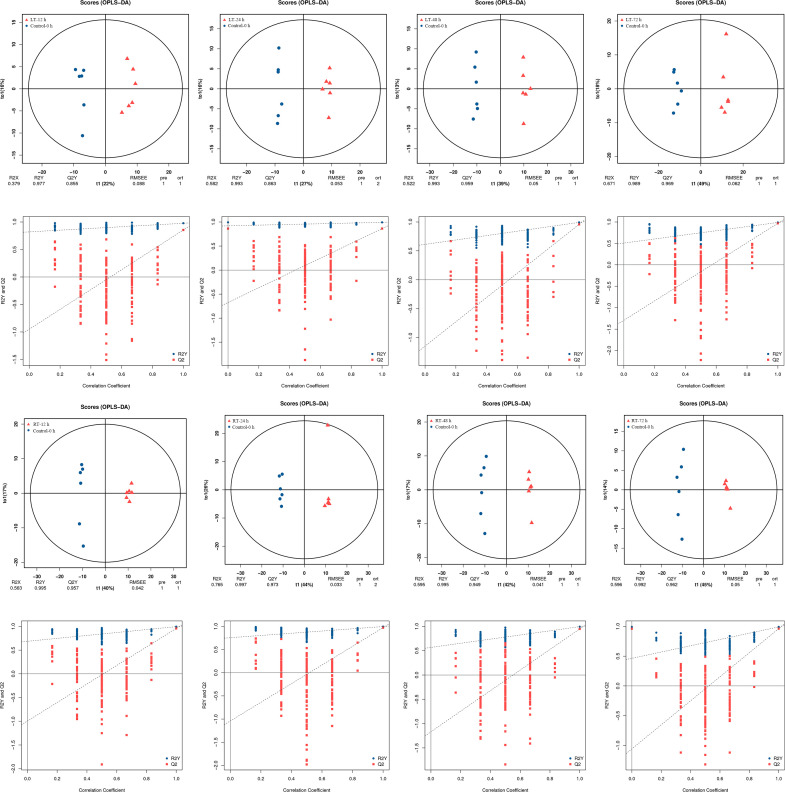
Metabolomic analysis of postharvest toon buds using GC-TOF-MS, including the OPLS-DA plots of RT, LT-stored samples, and control (top figure). Performance of the permutation tests validated from the OPLS-DA model (bottom figure).

The metabolic data between groups (control and 12−24 h of LT-stored groups, 48 h of RT-stored and 72 h of RT-stored groups, 48−72 h of LT-stored and 12−24 h of RT stored groups) had high correlation coefficients, indicating that these groups had similar metabolite accumulation patterns (**Supplementary Figure S1**). PCA was performed to reveal the distribution trends among various samples (**Supplementary Figure S1**). The differences between the samples could not be explained through the visual discrimination generated by the PCA. The orthogonal projections to latent structures-discriminant analysis (OPLS-DA) and permutation plots indicated the clear separation of the eight coupled treatments between control and other storage times (**Figure 3**). In general, an R2Y value of 0.65 or more and Q2Y of 0.5 or more indicated a satisfactory ability for quantitative prediction. High Q2, R2X, and R2Y values were observed in the comparison between control (0 h) and other samples. This observation indicated major time-dependent relationships in the metabolic profiles of postharvest toon buds.

### 3.5 DEMs in RT- and LT-stored toon buds compared with control

**Figure 4:**
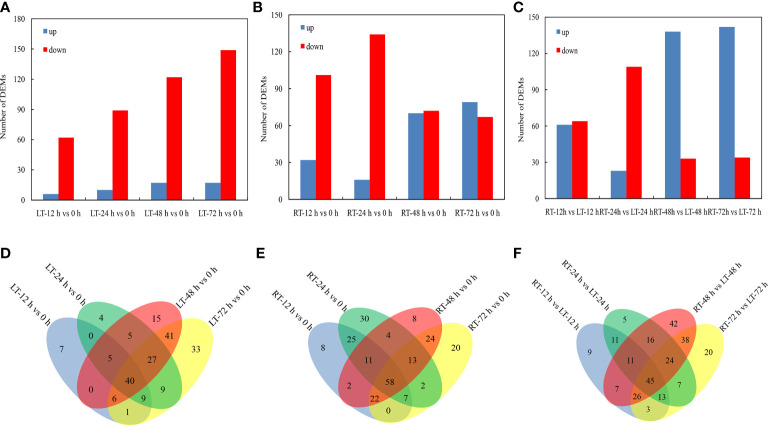
Distribution of DEMs at different storage times after harvest. Identification and number statistics of DEMs based on VIP > 1 and *P* < 0.05 **(A–C)**. According to Venn diagram analysis, the DEMs overlapped at different times under RT and LT storages after harvest **(D–F)**.

A differential metabolomics method based on VIP > 1 and *P* < 0.05 was used to screen the DEMs of postharvest toon buds. The total differential metabolites of LT vs. control, RT vs. control, and RT vs. LT at the same storage time point were 192 (**Figures 4A, D**), 234 (**Figures 4B, E**), and 277 (**Figures 4C, F**), respectively.

**Figure 5:**
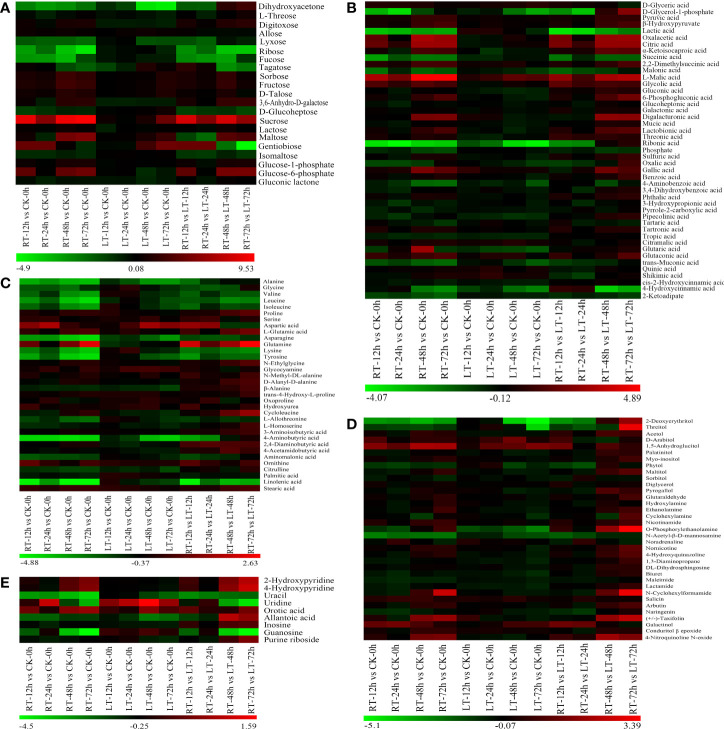
Hierarchical cluster analysis of changes in various carbohydrates **(A)**, organic acids **(B)**, amino acids **(C)**, alcohol, aldehyde, amine, and their derivatives **(D)**, and pyridine, pyrimidine and purine **(E)** in toon buds during storage. Red indicates high abundance. The metabolites with low relative abundance are shown in green. (The color key scale is at the bottom of the heatmap).

We built an HCA to unveil the diversity in the metabolite profiles among different experimental samples (**Figure 5**). Among the known differential metabolites, 16 carbohydrates (**Figure 5A**), 45 known acid metabolites (**Figure 5B**), 13 amino acids and their 18 derivatives (**Figure 5C**), 36 secondary metabolites (**Figure 5D**), and nine pyridines, pyrimidines, purines, and their derivatives (**Figure 5E**) were identified.

As shown in **Figure 5A**, the change patterns of various carbohydrates in toon buds under RT and LT storage were different. Sorbose, fructose, sucrose, lactose, D-talose, and maltose displayed greater upregulation in RT storage than in LT storage. The decrease in the relative contents of lysose, ribose, and fucose in toon buds during RT storage was more than that at LT storage. Digitoxose and 3,6-anhydro-D-galactose were upregulated under RT storage and downregulated under LT storage. D-Glucoheptose and isomaltose were downregulated under RT storage but not significantly.

As shown in **Figure 5B**, 16 organic acids related to glucose metabolism, including glucose 1-phosphate, glucose 6-phosphate, and 6-phosphogluconic acid, accumulated in different levels between LT and RT groups. The relative contents of inorganic sulfuric acid increased at RT, particularly after 48 h of storage. However, they decreased under LT storage. Phosphoric acid decreased rapidly during RT storage but changed slightly under LT storage.

Except for aspartic acid, glutamic acid, and glutamine, most amino acids and their derivatives exhibited a declining trend during storage. Moreover, the decrease in amino acid contents in the RT storage was greater than that in the LT storage. The relative contents of essential fatty acids such as palmitic acid and linolenic acid also showed a similar downward trend. However, the change in stearic acid was not remarkable during storage (**Figure 5C**).

Except for the primary metabolites, 36 secondary metabolites were classified into alcohol, phenol, aldehyde, amine, and their oxides (**Figure 5D**).

Given the catabolism of vitamins and uracil, 2-hydroxypyridine, 4-hydroxypyridine, and orotic acid accumulated significantly in toon buds during RT storage (**Figure 5E**). In contrast, purine and pyrimidine such as uracil, allantoic acid, hypoxanthine nucleoside, guanine nucleoside, and purine nucleoside decreased significantly during RT storage.

### 3.6 Differentially expressed genes related to the primary metabolism, shikimic acid, and ethylene biosynthesis

**Figure 6:**
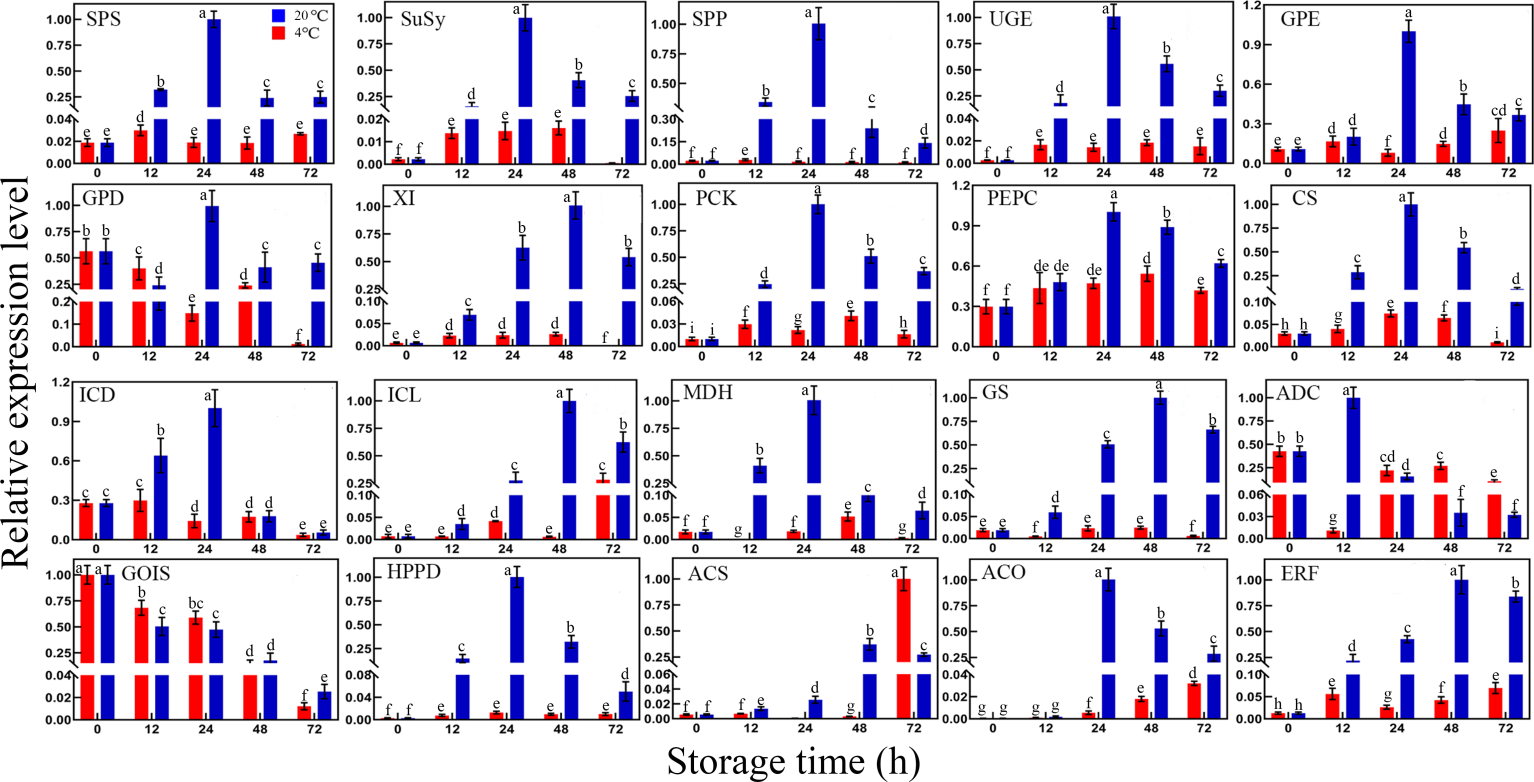
Differential expression of genes related to sucrose biosynthesis, sugar, organic acid, amino acid metabolism, ethylene biosynthesis, and signal transduction pathway in toon buds between RT and LT storages. SPS, sucrose-phosphate synthase; SuSy, sucrose synthase; SPP, sucrose-phosphatase; UGE, uridine diphosphate-glucose 4-epimerase; GPE, glucose-6-phosphate 1-epimerase; GPD, glucose-6-phosphate 1-dehydrogenase; XI, xylose isomerase; PCK, phosphoenolpyruvate carboxykinase; PEPC, phosphoenolpyruvate carboxylase; CS, citrate synthase; ICD, isocitrate dehydrogenase; ICL, isocitrate lyase; MDH, malate dehydrogenase; GS, glutamate synthase; ADC, arginine decarboxylase; GoLS, galactinol synthase; HPPD, 4-hydroxyphenylpyruvate dioxygenase; ACS, 1-aminocyclopropane-1-carboxylate synthase; ACO, 1-aminocyclopropane-1-carboxylate oxidase; ERF, ethylene-responsive transcription factor. The values are the means ± SEM from biological replicates. The bars with different lowercase letters are significantly different at *P* < 0.05.

As sucrose, shikimic acid, and ethylene accumulated significantly during storage, the expression levels of genes related to these metabolism pathways were further investigated (**Figure 6**). The expression levels of genes related to sucrose biosynthesis, including sucrose-phosphate synthase (*SPS*), sucrose synthase (*SuSy*), and sucrose-phosphatase (*SPP*) were significantly upregulated under RT storage. Three glucose and xylose isomerized-related genes, namely, uridine diphosphate-glucose 4-epimerase (*UGE*), glucose-6-phosphate 1-epimerase (*GPE*), and xylose isomerase (*XI*), play critical roles in monosaccharide configuration transformation during storage. Compared with the control, *UGE*, *GPE*, and *XI* were upregulated significantly, particularly after 24 h of storage. A group of genes related to gluconeogenesis and the TCA cycle was also analyzed. Compared with the control and LT storage, the expression levels of phosphoenolpyruvate carboxykinase (*PCK*), phosphoenolpyruvate carboxylase (*PEPC*), citrate synthase (*CS*), isocitrate dehydrogenase (*ICD*), isocitrate lyase (*ICL*), and *MDH* were obviously upregulated during RT storage. In particular, their relative expression levels reached the maximum after 24–48 h during RT storage. This finding indicated that gluconeogenesis was strengthened and the balance of the TCA cycle was disturbed within 24 or 48 h of RT storage. Oppositely, LT effectively suppressed gluconeogenesis and maintained the relative balance of the TCA cycle.

In addition to those genes related to sugar metabolism, two key enzyme genes involved in amino acid metabolism, including glutamate synthase (*GS*) and arginine decarboxylase (*ADC*), were observed. Similar to *ICL*, the *GS* expression level of RT storage was significantly higher than that of LT storage. The *GS* expression displayed an increasing trend with prolonged RT storage time. Unlike *GS* expression, *ADC* expression reached the maximum under 12 h of RT storage. Then, the *ADC* expression began to decrease gradually. Its expression level during RT storage was lower than that during LT storage. The expression level of galactinol synthase (*GoLS*) decreased gradually with the extended RT storage time. This finding was consistent with the relative content decrease of galactinol. Moreover, 4-hydroxyphenylpyruvate dioxygenase (*HPPD*), an essential enzyme gene involved in aromatic compound biosynthesis, significantly increased during RT storage. However, LT storage effectively inhibited *HDDP* expression. Thus, LT storage contributed to the reduction of the secondary acids containing aromatic metabolites and the preservation of postharvest toon bud quality.

As mentioned above, the ethylene release rate rapidly increased during RT storage. The expression patterns of the three genes involved in the ethylene signaling transduction pathway were further investigated. Two ethylene biosynthetic-genes, namely, 1-aminocyclopropane-1-carboxylate synthase (*ACS*) and 1-aminocyclopropane-1-carboxylate oxidase (*ACO*), and an ethylene-responsive transcription factor (*ERF*) were obviously upregulated during storage. Moreover, their expression levels at RT storage were much higher than those at LT storage. The results indicated that ethylene-triggered events in toon bud may happen after postharvest storage, leading to toon bud senescence and quality deterioration.

## 4 Discussion

### 4.1 Changes in the appearance and physiological characteristics of toon bud during storage at RT and LT

Toon buds are widely favored as delicious and nutritious vegetables by consumers. However, postharvest toon buds are susceptible to decay because of their active respiration and vigorous metabolism, resulting in the loss of nutritional value following quality deterioration during RT storage (Zhao et al., 2018; Jiang et al., 2019; Lin S. H. et al., 2019). LT storage can effectively inhibit the respiration of harvested toon buds, maintain their quality, and prolong their postharvest shelf life. Our results further verified that LT could reduce the respiration rate of toon buds and strongly repress ethylene release after harvest.

### 4.2 Changes in the sugar metabolism of toon buds during storage at RT and LT

The sugars with high sweetness, such as sucrose and fructose, accumulated rapidly with the prolonged storage time, particularly at RT storage. In comparison, the sugars with low sweetness related to stress showed a downward trend. This finding implied that the rot of toon buds was aggravated and the protective effect of osmolytes was weakened. Sugars are the main respiratory substrates for energy supply and osmolytes (Guo et al., 2019; Uarrota et al., 2019). Sucrose and fructose accumulated rapidly with the extended storage time. The possible reason was that the hydrolysis of starch provided glucose for sucrose synthesis. Alternatively, glucose 1-phosphate or glucose 6-phosphate flowed through the gluconeogenesis pathway to promote sucrose synthesis further (Farcuh et al., 2018; Luo et al., 2021). **Figure 6** shows that the expression levels of the key enzyme genes of sucrose synthesis increased rapidly during RT storage. However, their expression levels at LT storage were significantly lower than those at RT storage. The increase level of sucrose was quite limited under LT storage conditions. Many studies have shown that ethylene-dependent sucrose accumulation promoted postharvest ripening and senescence of fruits and vegetables (Foukaraki et al., 2015; Xu et al., 2016; Farcuh et al., 2018). Ethylene release rate increased during postharvest ripening of apples, resulting in sucrose and fructose accumulations (Sun et al., 2021). Exogenous ethylene application stimulated the expression of *SuSy* in postharvest blueberries (Wang et al., 2020). Our results were consistent with the above reports.

The intermediates’ accumulation in the glycolysis indicated that the metabolic pathway gradually weakened (Luo et al., 2021). The gene encoding glucose-6-phosphate 1-dehydrogenase (*GPD*) is the key enzyme of PPP metabolism (Perotti et al., 2015). During storage for 24−48 h, the *GPD* expression level at RT was significantly higher than that at LT. The results suggested that the PPP metabolism was enhanced in stored toon buds. The transcript level of the *XI* gene involved in the PPP metabolism also displayed an increasing trend at RT. Its expression was extremely low under LT storage, reflecting the strengthening of PPP metabolism under RT storage.

Previous studies have shown that the respiratory metabolism of good fruit and vegetable harvested was mainly based on EMP and TCA cycle pathways, and PPP was used as the auxiliary respiratory metabolism (Guo et al., 2019; Lin L. J. et al., 2019; Guo et al., 2022). However, PPP became the active respiratory metabolism for postharvest deteriorated fruit and vegetable tissues. In our results, the toon buds at the early stage of postharvest storage (0−24 h) were robust and healthy. Moreover, the respiratory pathway was mainly through the EMP-TCA cycle to provide sufficient energy for the life activities of the tissues. After 24 h of storage, the browning and quality deterioration of the harvested toon buds began, and the respiratory metabolism considerably depended on PPP.

### 4.3 Changes in amino acid and organic acid metabolisms of toon buds during storage at RT and LT

In addition, various essential amino acids decomposed severely with increasing storage time, resulting in a decrease in their contents. However, the levels of amino acids maintained at LT storage were higher than those at RT. For example, alanine and aspartic acid were transformed into pyruvate and oxaloacetic acid, respectively. Other amino acids, such as valine, leucine, isoleucine, and lysine, were transformed into α-ketoisocaproic acid through the combined transamination (Bekele et al., 2015; Ren et al., 2020). The *GS* and *ADC* in toon buds at RT storage exhibited significantly higher expressions than those at LT storage. This scenario further aggravated the accumulation of organic acids, such as oxaloacetic acid, citric acid, α-ketoisocaproic acid, and malic acid in the TCA cycle. However, this metabolic flow slowed down under the LT storage of toon buds. Many studies have shown that during postharvest storage, the upregulated expression of various enzyme genes in the TCA cycle accelerated respiratory consumption and reduced the contents of various organic acids (Lin et al., 2015; Liu et al., 2016; Yao et al., 2018). Our results did not exactly accord with previous reports. The possible reason was the upregulated expression of *PEPC* and *PCK* genes in the gluconeogenesis pathway, which converted pyruvate into oxaloacetic acid and reduced it to form malic acid (Perotti et al., 2015; Han et al., 2018).

The expression level of *ICL*, the key gene in the glyoxylic acid cycle in the bypass of the TCA cycle pathway, was also highly expressed under RT storage compared with LT storage. This finding indicated that the glyoxylic acid cycle involved in fatty acid catabolism was also strengthened. Intermediate accumulation in the EMP−TCA pathway strengthened gluconeogenesis, transforming non-sugar substances into sugars such as fructose and sucrose (Bekele et al., 2015; Liu et al., 2016).

### 4.4 Accumulation of aldehydes, acids, amines, and other compounds in toon buds during storage at RT and LT

The superfluous intermediates derived from sugars and amino acids, such as various organic acids, were metabolized into various toxic compounds, such as aldehydes, acids, and amines, through side reaction. This phenomenon further accelerated the quality deterioration of postharvest toon buds. However, this process slows down significantly under LT storage. Moreover, the relative levels of aldehydes, acids, amines, and their derivatives were significantly lower than those under RT storage. Phosphoenolpyruvate, the intermediate of gluconeogenesis pathway, combined with erythrose 4-phosphate, the intermediate of PPP pathway, to form other phenolic acids in the shikimic acid pathway (Becerra-Moreno et al., 2015). The upregulated expression of *HPPD* indicated that the transformation from primary metabolism to secondary metabolism was accelerated in toon buds. This result was consistent with our previous report (Zhao et al., 2021).

**Figure 7:**
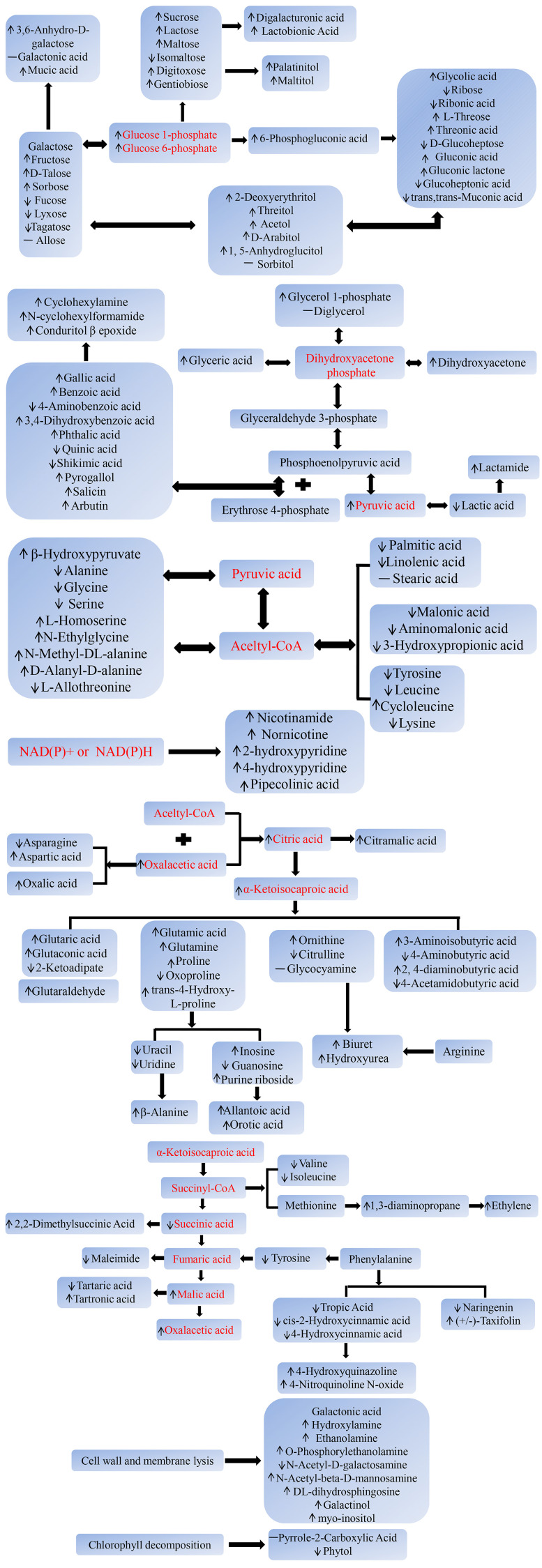
A summary of the significantly changed metabolic pathways in RT and LT storages. Narrow: upregulated or downregulated; dash: unchanged.

On the basis of the above results, we preliminarily described the changes in the molecular metabolic pathways of postharvest toon buds. As demonstrated in **Figure 7**, the sucrose metabolism, hexose isomerization, gluconeogenesis pathway, and PPP metabolic pathway that centered on glucose 1-phosphate or glucose 6-phosphate were strengthened. In comparison, the glycolysis pathway and the TCA cycle gradually weakened with the prolonged storage time. The increased catabolism of fatty acids and amino acids and the active side reactions, such as transamination, deamination, dehydrogenation, and oxidation, led to the accumulation of ketones, acids, aldehydes, and amines. The metabolic transformation of aromatic amino acids and the accelerated catabolism of pyrimidine or purine nucleotides led to toxic compound accumulation and cell energy consumption. All these circumstances led to the loss of nutrition and quality deterioration of toon buds after harvest.

## 5 Conclusions

In summary, the molecular mechanism of quality deterioration in toon buds was explored by comparatively analyzing the metabolic differences in toon buds between RT and LT storages. The results showed that compared with RT storage, LT storage could effectively inhibit the respiration rate and the ethylene release rate of toon buds. Moreover, the catabolism of various essential amino acids and fatty acids maintained a comparatively stable level. The expression levels of the key enzyme genes associated with sugar metabolism in LT-stored toon buds were significantly lower than those in RT-stored toon buds, resulting in the limited accumulation of sucrose and various organic acids. However, the primary metabolism displayed the opposite trend in the RT-stored toon buds. Compared with LT storage, RT storage promoted the metabolism of branched-chain synthesis pathways, such as shikimic acid, resulting in the accumulation of various aromatic compounds. In addition, various toxic compounds formed by oxidation, decarboxylation, and transamination rapidly increased under RT storage, eventually leading to quality deterioration and nutrition loss of postharvest toon buds.

## Data availability statement

The original contributions presented in the study are included in the article/**Supplementary Material**. Further inquiries can be directed to the corresponding author.

## Author contributions

HZ conceived and designed this experiment. HZ drafted the manuscript. CS and QH collected samples of toon sprouts. MF and XL extracted and assayed flavonoid components and volatile terpenoid compounds. JW carried out. qRT-PCR experiments and analyzed the data. All authors contributed to the article and approved the submitted version.

## Glossary

**Table:**

ACO	1-aminocyclopropane-1-carboxylate oxidase
ACS	1-aminocyclopropane-1-carboxylate synthase
ADC	arginine decarboxylase
CS	citrate synthase
EMP	Embden–Meyerhof pathway
ERF	ethylene-responsive transcription factor
GC	gas chromatograph
GC-TOF-MS	gas chromatograph coupled with a time-of-flight mass spectrometer
GoLS	galactinol synthase
GPD	glucose-6-phosphate 1-dehydrogenase
GPE	glucose-6-phosphate 1-epimerase
GS	glutamate synthase
DEMs	differentially expressed metabolites
DI	decay index
HCA	hierarchical clustering analysis
HPPD	4-hydroxyphenylpyruvate dioxygenase
ICD	isocitrate dehydrogenase
ICL	isocitrate lyase
IS	internal standard
LT	low temperature
MDH	malate dehydrogenase
MSSM	modified small-skep-method
OPLS–DA	orthogonal partial least squares discriminant analysis
PCA	principal component analysis
PCK	phosphoenolpyruvate carboxykinase
PEPC	phosphoenolpyruvate carboxylase
PPP	pentose phosphate pathway
qRT-PCR	quantitative real-time PCR
RSD	relative standard deviation
RT	room temperature
SPP	sucrose-phosphatase
SPS	sucrose-phosphate synthase
SuSy	sucrose synthase
UGE	uridine diphosphate-glucose 4-epimerase
VIP	variable importance in the projection
XI	xylose isomerase
