# Supplementary figures and images for: Metabolic profiling and gene expression analysis reveal the quality deterioration of postharvest toon buds between two different storage temperatures
Source: Front Plant Sci. 2023 Mar 20;14:1142840. doi: 10.3389/fpls.2023.1142840 (PMC10067724; doi:10.3389/fpls.2023.1142840)

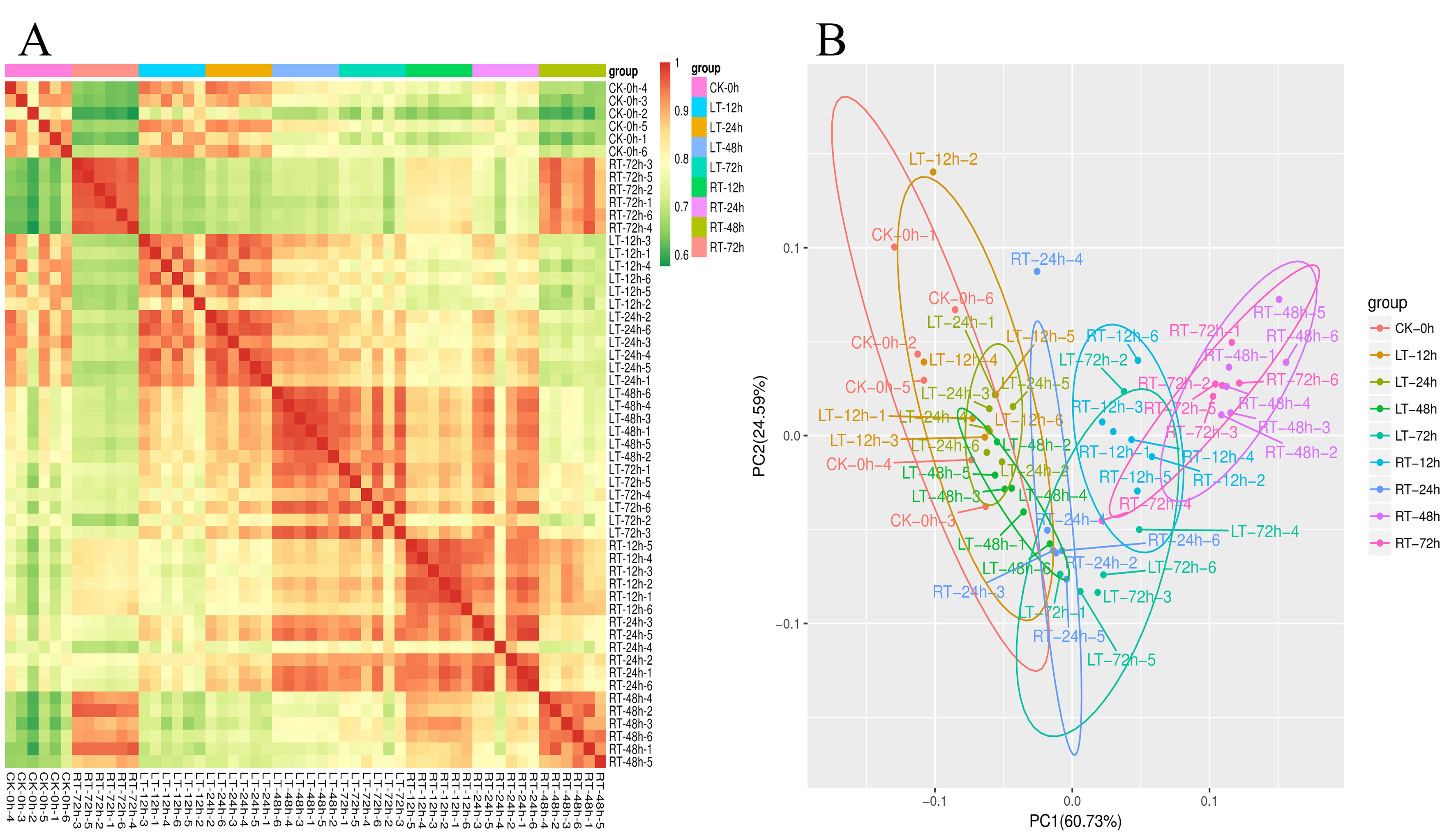

Supplement: Supplementary file 1 [file Image_1.tif]
